# Decreased expression of neuronal Per-Arnt-Sim domain protein 4 gene in the hippocampus of a post-stroke depression rat model

**DOI:** 10.3892/etm.2014.1537

**Published:** 2014-02-12

**Authors:** ZHAOHUI ZHANG, PENGGE FEI, JUNLIN MU, HAOLIANG WANG, WENQIANG LI, JINGGUI SONG

**Affiliations:** 1 Department of Psychosomatic Medicine, The Second Affiliated Hospital of Xinxiang Medical University, Xinxiang, Henan 453002, P.R. China; 2 Department of Neurology, The Second Affiliated Hospital of Xinxiang Medical University, Xinxiang, Henan 453002, P.R. China; 3 The Henan Key Lab of Biological Psychiatry, Xinxiang Medical University, Xinxiang, Henan 453002, P.R. China

**Keywords:** post-stroke depression, rat, hippocampus, neuronal Per-Arnt-Sim domain protein 4, expression

## Abstract

Neuronal Per-Arnt-Sim domain protein 4 (NPAS4) is important in regulating transcription and function in the limbic system and in brain development. Post-stroke depression (PSD) is a common complication following a stroke. Furthermore, organic damage as a result of a stroke affects the restoration of nerve function and indicates that hippocampal neural activity may be associated with PSD. A PSD rat model was established via a middle cerebral artery occlusion procedure, which was combined with isolation and chronic unexpected mild stress, and was used to investigate the expression of the NPAS4 gene in the hippocampus. The neurological deficit and behavior were evaluated and NPAS4 mRNA expression was measured by semi-quantitative reverse transcription-polymerase chain reaction; furthermore, the association with cognitive impairment was analyzed. The PSD rats displayed neuropsychopathic disorders and the NPAS4 mRNA expression levels in the hippocampus were significantly lower in the depression and PSD groups compared with the control group. Therefore, the present study identified that NPAS4 expression was decreased in the hippocampus of PSD rats.

## Introduction

Neuronal Per-Arnt-Sim domain protein 4 (NPAS4) is an alkaline basic helix-loop-helix (bHLH)-PAS transcription factor in the brain. NPAS4 is highly expressed in the hippocampus, where it forms a dimer with the hydrocarbon receptor nuclear translocation protein (1). It is important in regulating transcription and function in the limbic system as well as in brain development. In addition it influences the survival of neurons, cell protection in drug abuse, dendritic cell skeleton formation in the hippocampus, development and maintenance of synapses, transcription and regulation of synaptic plasticity, as well as being involved in the neuroendocrine system (2–3).

A cross-sectional epidemiology study in different brain regions demonstrated that post-stroke depression (PSD) was a common complication following a stroke, with an incidence rate ranging between 5 and 63% (4). PSD patients exhibited serious cognitive impairments, which were associated with mortality (5). Depression and cognitive impairment as a result of PSD aggravated the primary disease, hindering patient recovery and the healing process (6).

The hippocampus is the anatomical basis of learning, memory, and emotional and cognitive activities. The hippocampus and its adjacent temporal lobe participate in numerous learning and memory processes, regulated by dynamic changes in neurotransmitters (7,8). A previous study indicated that hippocampal nerve cell proliferation was reduced in PSD model rats during chronic stress, demonstrating that organic damage resulting from a stroke affects nerve function restoration, as well as indicating that hippocampal neural activity may be associated with changes in cognitive function in PSD patients (9).

As NPAS4 is significant in inhibitory post-synaptic development and stress-induced hippocampal damage (10), the changes in NPAS4 expression were investigated and the cognitive impairment was demonstrated by behavioral changes in the rat PSD model. The results of the present study may provide laboratory references for specific biological change indicators of PSD and increase the understanding of the pathogenesis of PSD and cognitive impairment.

## Materials and methods

### Animals

Specific-pathogen-free Sprague-Dawley rats (age, eight weeks; body weight, 160±20 g) were supplied by the Experimental Animal Center of Zhengzhou University (Henan, China). The animals were fed independently in a ventilated cage and provided with food and water *ad libitum* throughout the experiment. Room temperature was maintained at 22±2°C, with a natural circadian cycle. The animal experiments were conducted in accordance with the replacement, reduction and refinement principle to protect the welfare of the laboratory animals. The study was approved by the ethics committee of the Second Affiliated Hospital of Xinxiang Medical University (Xinxiang, China).

### Groups and models

The sucrose water consumption and open-field test (OFT) behavior scores were initially conducted to determine baseline values. Sixty mice with matching behavior scores were selected and randomly divided into control, depression and surgery groups (including a simple stroke group and a PSD group), respectively. Rats that succumbed as a result of surgery, during the stress process or due to other experimental factors were randomly replaced to ensure that there were six rats in each of the final groups, which were as follows: i) Control group, four rats per cage were subjected to a sham treatment, which was the same as the surgery group, without insertion of an embolization line into the carotid artery; ii) simple stroke group, a middle cerebral artery occlusion (MCAO) model was established via embolization. After 24 h, the Longa *et al* (11) method was used to evaluate the degree of neurological deficit on a scale of 0–4. Rats with a postoperative neurologic deficit score that was ≥1 and <4 were selected and housed (four rats per cage); iii) simple depression group, a chronic stress model of depression was established by exposure to chronic unpredictable mild stress (CUMS) using seven different stimuli (fasting, water deprivation, behavioral restriction, wet litter, electric shock to the foot, forced ice-water swimming and tail clamping) each of which were exerted four times, randomly distributed in four stress cycles and combined with isolation-housing; and iv) PSD group, MCAO rats were subjected to isolation-housing and CUMS (seven different stimuli each used four times, randomly distributed in four stress cycles, on day seven following surgery). The specific methods have been described in previous studies (12,13).

### Behavioral assessment

Body weight was measured prior to surgery as well as on days one, seven and 28 following surgery, to assess the changes. Sucrose water consumption was assessed and the proportion of 1% sucrose water that was consumed relative to normal water consumption, within 1 h after water deprivation for 24 h, was recorded prior to surgery and on days seven and 28 following surgery.

The OFTs were performed using an experimental device consisting of an open-field sound-proof response box (RWD, Shenzhen, China) and an automatic data-acquisition and processing system (Panlab, Barcelona, Spain). The behavior of each individual rat, regarding horizontal and vertical movements, was determined in the open-field response box over 5 min to measure any spontaneous activity, independent ability to explore and cognition. The assessments were conducted under quiet conditions, at appropriate and consistent levels of light intensity, temperature and humidity. The wall and bottom of the box were cleaned between each experiment to avoid the passage of information between the rats, for example through the scent of urine.

### Semi-quantitative reverse transcription-polymerase chain reaction (RT-PCR)

Total RNA was extracted and RT-PCR was performed according to the RNAiso Plus manual (Takara, Bio Inc., Shiga, Japan). Glyceraldehyde-3-phosphate dehydrogenase (GAPDH) served as a reference gene and the gene primers were designed based on the National Center for Biotechnology Information database (http://www.ncbi.nlm.nih.gov/, Table I). The cycle parameters for semi-quantitative RT-PCR were as follows: Denaturation at 95°C followed by a cycle of 3 min, 94°C for 15 sec and 68°C for 30 sec over 35 cycles, ending with a temperature of 15°C. A blank tube without cDNA template solution was subjected to PCR to serve as a negative control. A 5 μl volume of the PCR reaction product was mixed with 1 μl sample buffer and electrophoresed for 30 min at 180 V on 1.5% agarose gel. Images were obtained using an ultraviolet gel imaging system (Uvitec, Cambridge, UK) and the optical densities (ODs) of the electrophoretic bands were measured with GAS7001B gel image analysis software (Uvitec, Cambridge, UK) to indicate the quantity of PCR product. The expression level of the study gene was corrected to the reference gene, GAPDH, to demonstrate the relative strength of the gene expression according to the following formula: Relative expression quantity = target gene product OD/reference gene product OD.

### Statistical analysis

The data were analyzed using SPSS 13.0 statistical analysis software (SPSS Inc., Chicago, IL, USA). All of the measurements were presented as means ± standard deviation and assessed for normality. The groups were compared using one-way analysis of variance for pairwise comparisons in addition to using the least significant difference test. A two-tailed value of P<0.05 was considered to indicate a statistically significant difference.

## Results

### Effects on body weight

In the present study it was identified that in the composite PSD model a simple stroke led to initial weight loss and subsequent weight gain was slower. On day 28 following surgery, the body weights of the rats in the simple stroke, simple depression and PSD groups were significantly lower compared with the control group (P<0.01). Furthermore, the difference between the simple stroke and PSD group was identified to be significant (P<0.01), while the difference between the simple depression and the PSD group was not significant (P>0.05; Table II). Thus, body-weight loss was considered to reflect a neuropsychopathic condition.

### Sucrose water consumption

The simple depression (P<0.05) and PSD groups (P<0.01) exhibited lower consumption of sucrose water on day 28 post-surgery, compared with the control group. However, there was no difference identified between the simple stroke and control groups or between the simple depression and PSD groups (P>0.05; Table III). These results demonstrated the anhedonia that was exhibited by the simple depression and PSD groups.

### OFT

The simple depression and PSD groups exhibited less horizontal (P<0.01) and vertical motion (P<0.01) at day 28 post-surgery, compared with the control group (Tables IV and V). The activity decreases demonstrated a decline of neurological function, depression as well as cognitive impairment.

### Semi-quantitative RT-PCR

Total RNA was subjected to electrophoresis and demonstrated apparent 28S and 18S bands; the entire lane showed a diffuse-band type. The R-value of the total RNA, according to the spectrophotometer analysis, was between 1.8 and 2.0; therefore, the RNA extraction confirmed the gene expression analysis. In addition, the PCR amplified a single band with the expected molecular weight (247 bp for NPAS4 and 244 bp for GAPDH).

### Hippocampal NPAS4 mRNA levels

Compared with the control group, hippocampal NPAS4 mRNA levels were significantly lower in the simple depression (P<0.01) and PSD groups (P<0.05). However, there was no difference identified between the simple depression and PSD groups, or between the stroke and control groups (P>0.05; Table VI).

## Discussion

NPAS4 is a specific bHLH-PAS transcription factor in the brain that is selectively induced by the influx of Ca^2+^, which may be a negative feedback mechanism that maintains a homeostatic balance between excitation and inhibition (14). The low expression of NPAS4 mRNA affect the formation and function of the hippocampal neurons, result in a reduced hippocampal volume and leading to an impairment of hippocampus-dependent fear memory in mice (15). NPAS4 is involved in the hypoxic-ischemic brain damage response via regulatory activation and/or inactivation. Its expression is self-regulated via a feedback loop and is directly regulated by the duration of ischemia (16). In addition, NPAS4 and aryl hydrocarbon receptor nuclear translocator 2 (ARNT2) combine with the brain-derived neurotrophic factor (BDNF) promoter I, referred to as the transcription factor RasRE (bHLH-PAS response element), and thus are significant in nerve-excitability-dependent transcription (17). Previous studies identified that BDNF in the primary cultured rat hippocampus was a target gene for NPAS4, indicating that NPAS4 may be directly involved in the expression of BDNF. Furthermore, BDNF may regulate NPAS4 through numerous inhibitory synapses (18).

The results of the present study demonstrated that rats with simple depression or PSD exhibited reduced NPAS4 mRNA expression, gained less weight or lost weight, consumed a reduced quantity of sucrose water, and exhibited fewer OFT movements compared with the control rats. NPAS4 expression is induced rapidly within the central nervous system by numerous factors, including neuronal membrane depolarization in the initial stage of cerebral ischemia, hypoxia, Ca^2+^ influx resulting in a series of cascade reactions, and the ongoing pathological opening of glutamate receptors due to brain ischemia and hypoxia. NPAS4 combines with ARNT2 to regulate the expression of BDNF, with consequent neuroprotective effects on brain injury and ischemia. NPAS4 is gradually normalized via its own feedback loop, which may explain why BDNF mRNA expression is transiently increased following cerebral ischemia (19). In addition, NPAS4 alone exhibits a neuroprotective effect in the brain of rats following ischemic injury. Stroke damage and late chronic stress factors disrupt the physiological and psychological balance within the body, which is associated with decreased expression of a variety of growth factors (20). Reduced levels of NPAS4 weaken the action of BDNF in the hippocampus (21), which directly and indirectly impacts on the plasticity of the neurons, and the synaptic structure and function. This results in changes in neurotransmitters and affects neuronal survival, hippocampus dendrite cytoskeleton formation, the development and maintenance of inhibitory synapses and synaptic plasticity via the transcriptional regulation process, which subsequently results in decreased inhibitory synapse formation, hippocampal dysfunction, neuroendocrine disorder and cortical atrophy. In conclusion, NPAS4 may be an important regulatory transcription factor in the cognitive impairment process, which directly or indirectly affects cognitive function via BDNF. Reduced levels of NPAS4 mRNA in the hippocampus of PSD rats may be a significant factor in the biological mechanisms underlying cognitive impairment.

## Figures and Tables

**Table I:** Primer sequences.

	Primer sequence		
		
Gene	Forward	Reverse	Product size (bp)
NPAS4	5′-CCCAGCACTGCCACGTTCCC-3′	5′-CCCAGCACTGCCACGTTCCC-3′	247
GAPDH	5′-GGGCTCTCTGCTCCTCCCTCT-3′	5′-CCGTTGAACTTGCCGTGGGT-3′	244

NPAS4, neuronal Per-Arnt-Sim domain protein 4; GAPDH, Glyceraldehyde-3-phosphate dehydrogenase.

**Table II:** Changes in the body weights of the rats.

		Body weight (g)			
		
Group	Rats (n)	Before surgery	Day 1 post-surgery	Day 7 post-surgery	Day 28 post-surgery
Control	7	278.26±10.82	282.26±10.02	298.93±9.51	360.21±17.37
Simple stroke	7	283.43±14.47	256.29±14.89^a^	201.43±19.04^b^	304.86±33.67^b^
Simple depression	7	279.36±12.39	280.14±12.25	284.00±11.91	277.50±18.45^b^
PSD	7	285.86±28.17	254.50±36.23^a^	211.14±21.24^b^	257.57±33.12^b^,^c^
F-value		0.274	3.507	66.162	19.348
P-value		0.843	0.031	<0.001	<0.001

Data are presented as means ± standard deviation.

a P<0.05 and

b P<0.01 compared with the control group;

c P<0.01 compared with the simple depression group.

PSD, post-stroke depression.

**Table III:** Levels of sucrose water consumption.

		Sucrose water consumption (g/100 g body weight)		
		
Group	Rats (n)	Before surgery	Day 7 post-surgery	Day 28 post-surgery
Control	7	8.20±0.96	7.96±0.87	6.53±0.76
Simple stroke	7	8.01±0.75	4.44±2.47^a^	7.05±0.56
Simple depression	7	8.29±0.93	7.64±1.45	4.56±1.59^b^
PSD	7	7.91±1.01	5.32±2.23^a^	4.67±1.34^b^
F-value		0.243	6.025	8.709
P-value		0.865	0.003	<0.001

Data are presented as means ± standard deviation.

a P<0.05 and

b P<0.01 compared with the control group.

PSD, post-stroke depression.

**Table IV:** Open-field test of horizontal movement.

		Horizontal movement (cm/5 min)		
		
Group	Rats (n)	Before surgery	Day 7 post-surgery	Day 28 post-surgery
Control	8	10599.76±774.97	10872.49±1227.55	10437.43±1936.89
Simple stroke	8	9508.60±1928.66	2636.93±755.01^a^	11011.57±1010.43
Simple depression	8	10951.67±2078.88	10781.93±2006.26	6584.53±4803.91^b^
PSD	8	10484.94±1430.58	2500.87±1318.45^a^	6806.53±3826.77^b^
F-value		1.000	81.203	3.607
P-value		0.410	<0.001	0.028

Data are presented as means ± standard deviation.

a P<0.05 and

b P<0.01 compared with the control group.

PSD, post-stroke depression.

**Table V:** Open-field test of vertical motion.

		Vertical movement (cm/5 min)		
		
Group	Rats (n)	Before surgery	Day 7 post-surgery	Day 28 post-surgery
Control	8	18.86±3.80	19.71±1.25	19.57±8.38
Simple stroke	8	20.71±5.44	7.71±4.35^a^	20.57±4.16
Simple depression	8	21.57±5.62	21.43±3.46	10.29±10.73^b^
PSD	8	19.00±2.38	7.43±6.16^a^	8.86±4.30^b^
F-value		0.606	22.612	4.714
P-value		0.618	<0.001	0.010

Data are presented as means ± standard deviation.

a P<0.05 and

b P<0.01 compared with the control group.

PSD, post-stroke depression.

**Table VI:** Expression of NPAS4 mRNA in the hippocampus.

Group	Rats (n)	NPAS4 mRNA
Control	7	1.01±0.19
Simple stroke	7	1.12±0.29
Simple depression	7	0.69±0.10^b^
PSD	7	0.74±0.08^a^
F-value		9.172
P-value		<0.001

Data are presented as means ± standard deviation.

a P<0.05 and

b P<0.01 compared with the control group.

NPAS4, neuronal Per-Arnt-Sim domain protein 4; PSD, post-stroke depression.
